# Younger age at surgery and lesser seizure frequency as prognostic factors for favorable seizure-related outcome after glioma resection in adults

**DOI:** 10.18632/oncotarget.18726

**Published:** 2017-06-27

**Authors:** Zhe-Ren Tan, Xiao-Yan Long, Zhi-Quan Yang, Jun Huang, Qing-Yuan Hu, Hao-Dong Yang, Guo-Liang Li

**Affiliations:** ^1^ Department of Neurology, Xiangya Hospital, The Central South University, Changsha 410008, China; ^2^ Department of Neurosurgery, Xiangya Hospital, The Central South University, Changsha 410008, China; ^3^ Ya Li High School, Changsha 410005, China

**Keywords:** glioma, brain tumor, epilepsy, prognostic factor, glioma resection

## Abstract

The identification of variables predictive of good seizure control following surgical tumor resection in adult glioma patients with tumor-related epilepsy would greatly benefit treatment decisions. Therefore, we analyzed the clinical data of adult patients with tumor-related epilepsy who underwent tumor resection at our institute between November 2011 and August 2013. Patients were divided into seizure-free (Engel Ia) and unfavorable outcome groups (Engel Ib–IV), and potential prognostic factors were analyzed. Of 90 patients, 61 (68%) had a favorable outcome at an average of 3 years after surgery. Our analyses indicated that younger age at surgery (P=0.048) and rare seizure frequency (P=0.006) were associated with significantly more favorable postoperative seizure-related outcomes. In conclusion, younger age at surgery and lesser seizure frequency were independent predictors of favorable epileptic seizure control after glioma resection in adults. Thus, early surgical resection is necessary for achieving favorable seizure outcome.

## INTRODUCTION

An estimated 50% of glioma patients develop tumor-related epilepsy [1–3], and seizure is often an initial symptom for patients with glioma [4]. The primary goals of tumor resection are to eliminate seizure activity and improve patients’ survival. Unfortunately, tumor resection does not have the desired effects in a significant percentage of glioma patients. Therefore, treatment planning for glioma patients would benefit greatly from the identification of prognostic factors for predicting the likelihood of seizure control following tumor resection. Most previous studies have focused on factors related to the survival of glioma patients, such as previous history of epilepsy, ethnicity, tumor location, age at diagnosis, extent of removal, tumor grade, and total tumor/edema volume [5–7]. Only a few studies have conducted multivariate analyses for seizure outcome following glioma resection, and the statistical results are controversial [8–12]. There are great differences between pediatric and adult patients in pathological type and treatment, and thus, we focused on the adult population. In the present study, we investigated the seizure outcomes of adult glioma patients to determine prognostic factors related to seizure control following surgical tumor resection.

## RESULTS

A total of 90 patients (47 men and 43 women) with glioma-related epilepsy were included in this study. All of the patients had a histological diagnosis of glioblastoma, astrocytoma, oligodendroglioma, or mixed glioma. The majority of patients (95.6%, 86/90) underwent complete gross total resection. No patients required a second surgery, and 79 patients with a WHO Grade II-IV glioma accepted postoperative radiochemotherapy, which included radiotherapy and temozolomide. All patients underwent at least a course (2.5 years) of antiepileptic drug (AED) therapy. The characteristics of all cases are listed in Table 1.

**Table 1:** Characteristics of glioma patients with tumor-related epilepsy

Variable	Value
**Gender, M/F**	47/43 (52%/48%)
**Follow-up period, years**	4.05±0.69 (3–5.7)
**Age at seizure onset, years**	36.12±11.07 (16–68)
**Age at surgery, years**	37.40±10.51 (17–68)
**Time from seizure onset to surgery, years**	1.29±1.79 (0.01–7.00)
**Site of surgery**	
**Left side**	51 (56.7)
**Right side**	39 (43.3)
**Extent of tumor removal**	
**Gross total resection**	86 (95.6)
**Partial resection**	4 (4.4)
**Main location of resection**	
**Temporal lobe**	38 (42.2)
**Frontal lobe**	35 (38.9)
**Parietal lobe**	9 (10.0)
**Occipital lobe**	4 (4.4)
**Basal ganglia**	4 (4.4)
**Tumor grade**	
**Class I**	11 (12.2)
**Class II**	67 (74.4)
**Class III**	8 (8.9)
**Class IV**	4 (4.4)
**Seizure type**	
**SPS**	23 (25.6)
**CPS**	31 (34.4)
**GS**	36 (40.0)

Data are n (%) or mean ± standard deviation (range).

M/F, male/female; SPS, simple partial seizures; CPS, complex partial seizures; GCTS, generalized seizures.

After a follow-up of 3 years, 61 patients (67.8%) were seizure free (Engel Class Ia), and 44 patients (48.9%, 44/90) were still seizure free after treatment and withdrawal of AED during the follow-up period. Whereas the other 29 patients had unfavorable outcomes categorized as Engel Class Ib–IV. No surgical complications were identified. Table 2 summarizes the year-by-year surgical outcomes of all patients. In a univariate analysis comparing the seizure-free group with the unfavorable outcome group, no significant differences were found in gender, age at seizure onset, time from seizure onset to surgical treatment, site of surgery, main location of surgery, tumor grade, chemoradiotherapy, duration of seizures, and seizure type. By contrast, rank sum test confirmed that a significant difference was detected in seizure frequency between the two groups (Table 3), and Row × Column contingency table and χ^2^ analysis confirmed that a significant difference was detected in age at surgery and seizure frequency between the two groups (Tables 4 and 5).

**Table 2:** Year-by-year seizure-related outcomes according to Engel class

Follow-up duration	Patients, n (%)	
	Class Ia	Classes Ib–IV
**6 months**	60/90 (66.7%)	30/90 (33.3%)
**2 years**	60/90 (66.7%)	30/90 (33.3%)
**3 years**	61/90 (67.8%)	29/90 (32.2%)
**4 years**	39/57 (68.4%)	18/57 (31.6%)
**5 years**	11/14 (78.6%)	3/14 (21.4%)

**Table 3:** Results of univariate analysis identifying variables that differed significantly between the seizure-free and unfavorable outcome groups

Quantitative variable	Seizure-free (n=61)	Unfavorable (n=29)	P-value
**t-test**			
**Age at onset (years)**	35.07±11.44	38.34±10.07	0.191
**Age at surgery (years)**	36.56±10.63	39.34±10.20	0.272
**Time from seizure onset to surgical treatment (years)**	1.44±2.01	1.00±1.20	0.201
**Rank sum test**			
**Duration of seizures (sec)**	179.70±160.63	268.28±271.86	0.289
**Seizure frequency (n/month)**	5.45±11.50	9.44±21.55	**0.022**

Quantitative results are expressed as the mean ± standard deviation.

**Table 4:** Analysis identifying qualitative variables that differed significantly between the seizure-free and unfavorable outcome groups

Qualitative variables	Seizure-free (n=61)	Unfavorable (n=29)	P-value
**Time from seizure onset to surgery ≤1 year**	37	17	0.854
**Seizure frequency (rare)**	37	8	**0.034**
**Site of surgery (left)**	34	17	0.796
**Gender (male)**	31	16	0.699
**Chemoradiotherapy**	55	24	0.319

Pearson's χ^2^ continuity correction of Fisher's exact test was used for statistical analysis.

**Table 5:** Row × Column contingency table and χ^2^ analysis identifying variables that differed significantly between the seizure-free and unfavorable outcome groups

Qualitative variables	Seizure-free (n=61)	Unfavorable (n=29)	P-value
**Age at surgery**			**0.006**
**≤32 years**	21	8	
**33–42 years**	28	6	
**>42 years**	12	15	
**Seizure type**			0.661
**SPS**	14	9	
**CPS**	21	10	
**GS**	26	10	
**Tumor grade**			0.611§
**WHO grade I**	6	5	
**WHO grade II**	47	20	
**WHO grade III-IV**	8	4	
**Main location of resection**			0.676§
**Temporal**	24	14	
**Frontal**	24	11	
**Parietal**	6	3	
**Others**	7	1	

RxC contingency tables and χ^2^ tests were used for statistical analysis. SPS, simple partial seizures; CPS, complex partial seizures; GS, generalized seizures;§:fisher’s exact test.

Tables 4 and 5 summarize the results of further analyses comparing the quantitative and qualitative variables between the seizure-free and unfavorable outcome groups. Multiple logistic regression analysis confirmed that age at surgery and seizure frequency were pre-surgical risk factors correlating with the postoperative seizure outcome (Table 6).

**Table 6:** Results of backward stepwise multiple logistic regression analysis of variables that differed significantly between the seizure-free and unfavorable outcome groups

Variable	Engel classification	P-value
	Regression coefficient	
**Age at surgery**	0.611	**0.048**
**Seizure frequency (rare)**	1.369	**0.006**
**Constant**	-4.130	0.000

## DISCUSSION

The objective of our study was to identify prognostic factors for seizure-related outcome following tumor resection in adult patients with glioma-related epilepsy, according to the Engel standard classification. Previous studies have reported that better seizure control was achieved in patients with a younger age at surgery and in cases with a shorter time period between seizure onset and surgical intervention [13]. Consistently, logistic regression analysis demonstrated that age at surgery was significantly associated with becoming seizure free after tumor resection in our study population. The previous study that identified a shorter period of seizure activity prior to surgical resection as a positive predictor of seizure control did separate patients according to the grade of glioma [8]. Unlike previous studies, our analyses did not identify the time from seizure onset to surgical treatment as significantly predictive of postoperative outcome. However, the effect of this time interval may have been lost due to the heterogeneity of the glioma types in the two patient groups, which included both low-grade and high-grade gliomas. Thus, the significance of the pre-surgical duration of seizure activity requires further analysis in larger cohorts of patients.

One previous study investigated the effect of the preoperative seizure frequency on postoperative seizure control and found a trend for improved outcomes with a lesser seizure frequency [14], and a remarkable finding in our study was the seizure-free status of a high proportion of patients in the rare seizure group (82.2%, 37/45 vs. 53.3%, 24/45 in the frequent seizure group). Whether a lesser preoperative epilepsy frequency is a positive prognostic factor associated with the elimination of seizures upon surgical resection continues to be debated.

The frontal, temporal, and parietal lobes are often considered as the regions with the greatest epileptogenic potential in tumor-related epilepsy [15, 16]. Consistently, in our study, the majority of gliomas were located in the temporal (42.2%), frontal (38.9%), and parietal (10.0%) lobes. However, our analyses did not identify the location of the tumor as a significant prognostic factor for seizure-related outcome.

In previous studies that compared patients with high-grade and low-grade gliomas, those with low-grade gliomas tended to have more epileptic seizures [1-4], and we believe this also occurred in our patient population. The previous studies showed that a low level of adenosine A1 receptor/adenosine A2a receptor expression, which was observed in low-grade gliomas, could increase susceptibility to tumor-related epilepsy [17]. In addition, increased expression of RAD50 interactor 1 (RINT1) and isocitrate dehydrogenase 1 (IDH1) R132H may represent risk factors for low-grade glioma-related seizures [18, 19]. However, tumor grade has not been reported to be a predictor of longitudinal prognosis after surgical tumor resection [12]. In the present study, no significant difference was found in tumor grade. Thus, whether tumor grade has an effect on the seizure-related outcome after resection is open for debate and further research.

The current study is limited by its single-center design, small sample size, and the heterogeneity of glioma type in the patient groups. Although we found that both younger age at surgery and lesser seizure frequency were predictive of favorable outcome, it is possible that an interaction exists between these variables that could influence our results. A multicenter, prospective or randomized controlled clinical trial in which all variables can be tracked and interactions can be tested would be expected to yield more reliable results.

Surgical resection is effective at achieving good rates of seizure freedom in patients with tumor-related epilepsy, and early age at surgery and lesser seizure frequency improve the likelihood of successful postoperative seizure control in adult. Although our analyses did not identify presurgical epilepsy duration as relevant to a favorable seizure-related outcome, prior research still indicates that this is beneficial for achieving favorable outcomes and survival.

## MATERIALS AND METHODS

We retrieved the clinical data for glioma patients treated between November 2011 and August 2013 in the Department of Neurosurgery, Xiangya Hospital, Central South University, Changsha, China. In each case the diagnosis of glioma was confirmed histologically. Seizure patterns were classified according to the International League Against Epilepsy (ILAE) classification system [20]. Patients were regarded as having epilepsy if they had a history of epilepsy or if they suffered at least one seizure during the diagnosis and treatment of the tumor.

We included only patients above the age of 14 years with tumor-related epilepsy and analyzed these cases retrospectively by screening the patients’ charts, an integral medical record, preoperative evaluation, and the results of postoperative follow-up (period ≥3 year). All patients underwent at least a course (2.5 years) of antiepileptic drug (AED) therapy with oxcarbazepine, and the dose was gradually decreased until the withdrawal of medication once the patient had been completely seizure free for 2 years. Patients were excluded if they had seizures that occurred during the course of the disease but were credited to other etiologies, such as intracranial infection or toxic-metabolic causes and subdural hemorrhage before the study began. Patients also were excluded if they had experienced glioma recurrence after tumor resection, which could influence postoperative seizure outcomes [21, 22]. The postoperative seizure outcome after resection was assessed during outpatient visits and by telephone contact. The position of the glioma was determined on preoperative magnetic resonance imaging (MRI) scans in all cases. The operation patterns included partial or gross total resection and extent of resection as evaluated by preoperative investigations and intraoperative adjuncts, such as contrast-enhanced MRI, T2 or FLAIR signal, DWI and PET, ultrasound navigation and 5-ALA, and intraoperative motor mapping and monitoring, etc. Postoperative glioma progression was monitored by repeated MRI every 6 months. Chemoradiotherapy was regarded as complete in patients who received at least one course of radiotherapy with a full cycle of doses between 45 and 60 Gy and at least one course (1 month) of chemotherapy with temozolomide. All patients were enrolled according to the principles of medical ethics, and written informed consent was obtained from each patient. Xiangya Hospital granted ethical approval for this study.

Postoperative seizure outcome was graded according to the method of the Engel standard classification [23]. In our study, seizure outcome was divided into “seizure free” (class Ia) or “unfavorable” (classes Ib-IV). The influence of each of the following patient characteristics on surgery outcome was analyzed: gender, age at seizure onset, age at surgery, time from seizure onset to surgical treatment (i.e., presurgical epilepsy duration), types of seizures experienced before resection, seizure duration, seizure frequency, site of surgery (left vs. right), main location of surgery (temporal lobe, frontal lobe, parietal lobe, others), chemoradiotherapy, and tumor grade according to World Health Organization (WHO) Grading of Tumours of the Central Nervous System from 2007 [24]. Seizure frequency was categorized as ‘‘frequent seizure’’ if the patient experienced seizure daily, weekly, or monthly and as “rare seizure’’ if the patient experienced seizure annually, sporadically, or only once.

Descriptive and frequency statistical analyses were performed using SPSS 18.0 for Windows (SPSS Inc., Chicago, IL, USA). The quantitative variables included age at seizure onset, age at surgery, pre-surgical epilepsy duration, duration of individual seizures, and seizure frequency per month prior to surgery, and the significance of these variables were tested by using two-sample t-test or non-parametric test. The qualitative variables included gender, time from seizure onset to surgery, seizure frequency, and chemoradiotherapy, and the significance of these variables was tested using Pearson’s χ^2^ or Fisher exact test. The significance of age at surgery, seizure type, WHO tumor grade, and main location of surgery were tested using Row × Column contingency table χ^2^ analysis. Finally, backward stepwise multiple logistic regression analysis was performed to predict surgical outcome according to potential prognostic factors identified in the first analyses. A P-value <0.05 was considered statistically significant.
